# Analysis of Putative CzcR Targets Under Zinc Sufficiency and Zinc Excess Conditions in *Pseudomonas aeruginosa* Using ChIP-Seq

**DOI:** 10.3390/microorganisms14050943

**Published:** 2026-04-22

**Authors:** Florian Mauffrey, Verena Ducret, Catarina Gonçalves Milho, Karl Perron

**Affiliations:** 1 Microbiology Unit, Department of Plant Sciences, Sciences III, University of Geneva, 1211 Geneva, Switzerlandkarl.perron@unige.ch (K.P.); 2 Institute of Pharmaceutical Sciences of Western Switzerland, University of Geneva, 1211 Geneva, Switzerland; 3 Section of Pharmaceutical Sciences, University of Geneva, 1211 Geneva, Switzerland

**Keywords:** *Pseudomonas aeruginosa*, zinc, ChIP-seq, CzcRS, regulon

## Abstract

*Pseudomonas aeruginosa* is a versatile opportunistic pathogen that thrives in hostile environments by tightly regulating zinc (Zn) homeostasis. The CzcRS two-component system is pivotal for Zn resistance, primarily by activating the CzcCBA efflux pump, yet its basal activity and full regulon remain poorly defined. Here, we analyzed putative CzcR targets under zinc sufficiency (ZS) and zinc excess (ZE) conditions in *P. aeruginosa* PAO1 using ChIP-seq. Under ZE, we identified 32 CzcR binding sites, potentially regulating 39 genes, many of which are linked to virulence, antibiotic resistance, and stress response. Under ZS, 10 binding sites were detected, revealing distinct CzcR targets. Considering the presence of a CzcR binding motif close to the peaks summit and RNA-seq data, we identified seven and four novel CzcR-regulated genes under ZE and ZS conditions, respectively, mostly implicated in bacterial virulence. Our findings highlight that CzcR may exhibit different functionalities depending on Zn concentration: its basal activity maintains physiological robustness, while its activated form orchestrates Zn detoxification and virulence modulation. This study expands our understanding of how *P. aeruginosa* integrates metal sensing with clinically relevant phenotypes, highlighting CzcR as a key regulator at the intersection of metal homeostasis and pathogenicity.

## 1. Introduction

*Pseudomonas aeruginosa* is a highly adaptable opportunistic pathogen responsible for severe infections, particularly in immunocompromised and cystic fibrosis patients [1,2]. Its success as a pathogen stems from its ability to survive and thrive in diverse and often hostile environments by producing a broad range of virulence factors and efficiently adjusting to fluctuations in essential trace metals such as Zn^2+^ [3,4,5]. Zinc (Zn) is the second most abundant transition metal in biological systems after iron and acts as a cofactor for numerous enzymes and transcription factors [6]. However, while essential at trace levels, Zn becomes toxic when in excess due to mismetallation, a phenomenon in which Zn^2+^ displaces other metals from metalloproteins, disrupting their function [7].

To cope with both Zn limitation and overload, *P. aeruginosa* has evolved a sophisticated and tightly regulated Zn homeostasis network composed of uptake, storage, and efflux systems [8,9]. This network allows the bacterium to maintain intracellular Zn concentrations within narrow physiological limits while rapidly adapting to host-imposed metal stress. Under Zn-deficient conditions, *P. aeruginosa* expresses high-affinity uptake systems, including several ABC transporters such as ZnuABC or PA4063–66, the pseudopaline-mediated ZrmABCD zincophore pathway, and the HmtA P-type ATPase. Under these Zn limited conditions, the bacterium can also release Zn by replacing C+ ribosomal or regulatory proteins by C − paralogs, even though this storage mechanism remains debated [9,10,11]. Conversely, under Zn excess (ZE), the bacterium induces export systems that are the P-type ATPase CadA, the cation diffusion facilitators CzcD and YiiP, and the tripartite CzcCBA efflux pump. This complex belongs to the Heavy Metal Efflux Resistance Nodulation Division superfamily and ensures efficient detoxification of the cytoplasm and periplasm [4,12,13]. Altogether, this network provides *P. aeruginosa* with the capacity to withstand extreme Zn fluctuations while enabling rapid transitions between Zn starvation and Zn excess states.

Three major regulators orchestrate this Zn homeostasis network. Zur represses Zn uptake systems when intracellular Zn reaches sufficient levels. Two regulators activate Zn export systems: CadR (also called ZntR), a MerR-family regulator that, upon metal binding, induces *cadA* transcription by modulating promoter conformation [14], and the two-component system (TCS) CzcRS, which plays a pivotal role in Zn resistance by inducing the highly efficient CzcCBA efflux pump [15]. CzcRS is composed of CzcS, a membrane-embedded histidine kinase capable of sensing excess periplasmic Zn^2+^ or Cd^2+^ and the response regulator CzcR. Crystal structures of the N-terminal periplasmic domain of CzcS demonstrated direct metal sensing without accessory proteins [16]. Metal binding triggers CzcS dimerization and autophosphorylation on a conserved histidine, followed by phosphotransfer to CzcR. Once activated the regulator binds target promoters and induces transcription of both *czcCBA* and *czcRS*, establishing a positive feedback loop. Importantly, proper activation of CzcRS requires Zur and CadA/CadR modules, revealing interdependence among Zn homeostasis regulation in *P. aeruginosa* [8,17].

Beyond its canonical role in metal export, CzcR exerts broader regulatory control over antibiotic resistance, virulence, and motility [18]. Specifically, CzcR represses transcription of *oprD*, a gene coding for the porin required for uptake of basic amino acids and carbapenem antibiotics, thereby contributing to Zn-dependent carbapenem resistance [19]. Consistently, carbapenem-resistant clinical and veterinary isolates frequently carry mutations in *czcS* or *czcR*, leading to constitutive *oprD* repression and increased carbapenem resistance [15,20,21]. Moreover, CzcR modulates biofilm formation, and virulence factors expression [18,22,23], making it a global regulator whose full influence remains unresolved.

In a general scheme, when TCS is active, the phosphorylated dimerized response regulator binds specific promoter sequences and acts as an activator, a repressor, or, as in the case of CzcR, a dual regulator [24,25,26]. Under Zn sufficiency (ZS) conditions, CzcR is undetectable, and transcript levels are minimal [19]. However, within the CzcR regulon, certain targets depend on basal CzcR levels and do not require Zn-induced activation. This may reflect phosphorylation-independent DNA binding, heterodimerization with partner regulators, cross-phosphorylation between TCS sensors, or even low-level spontaneous phosphorylation via acetyl phosphate.

Most studies on CzcR have focused on ZE conditions, where it is fully activated, and little is known about its activity under basal ZS conditions. A recent analysis identified additional CzcR targets by ChIP-seq and revealed regulatory overlap with BfmR, indicating co-regulation of several genes [27]. However, the genome-wide CzcR binding profile under different Zn conditions remains incompletely characterized. In this study, we aimed to refine the mapping of the CzcR regulon in *P. aeruginosa* under both ZS and ZE conditions. Using a Δ*czcRS* mutant as a control for our ChIP-seq analyses, we ensured maximal confidence in peak identification.

## 2. Materials and Methods

### 2.1. Bacterial Strains and Growth Conditions

Wild-type *P. aeruginosa* PAO1 (laboratory collection) and the double ∆*czcRS* mutant [28] were grown at 37 °C, under agitation, in solid or liquid Luria–Bertani (LB) medium (US biological, Salem, MA, USA). To constitute the ZE medium, LB medium was supplemented with 0.5 mM of ZnCl_2_. This concentration is sufficient to induce the Zn resistance response of the bacteria without causing a drastic growth defect [18,19].

### 2.2. Chromosome Immunoprecipitation (ChIP), ChIP-Seq Library Construction and Sequencing

The ChIP procedure was performed as previously described [29]. Three overnight cultures of *P. aeruginosa* WT or the Δ*czcRS* mutant were diluted to a final OD_600_ of 0.05 in 3 × 50 mL LB medium alone or supplemented with 0.5 mM ZnCl_2_. Cultures were incubated for 6 h at 37 °C with shaking, adjusted to the same OD_600_, and cross-linked with 1.2% formaldehyde for 10 min at 37 °C. Cross-linking was quenched with 330 mM glycine, followed by incubation for 5 min at room temperature and 5 min on ice. Cells were washed twice with ice-cold TBS to remove residual formaldehyde.

Pellets were resuspended in 0.6 mL FA lysis buffer (50 mM HEPES-KOH pH 7.5, 140 mM NaCl, 1 mM EDTA, 1% Triton X-100, 0.1% sodium deoxycholate) supplemented with 5 mg/mL lysozyme. PMSF (Thermo Fisher, Waltham, MA, USA) was added to 1 mM, and cells were lysed by sonication in the presence of 0.5% SDS, followed by centrifugation for 5 min at 4 °C. The supernatant was collected, and 30 µL of each extract was diluted in 370 µL TE, pH 8.0, and kept as input.

For immunoprecipitation, 100 µL of the extract was diluted in 800 µL FA lysis buffer and incubated overnight at 4 °C on a rotating wheel with 30 µL protein A magnetic beads (Invitrogen, Waltham, MA, USA, 10002D) and 6 µL polyclonal anti-CzcR antibody [30]. The beads were washed sequentially (1× each) with 1 mL FA lysis buffer, 1 mL FA500 buffer (50 mM HEPES-KOH pH 7.5, 500 mM NaCl, 1 mM EDTA, 1% Triton X-100, 0.1% sodium deoxycholate), 1 mL buffer III (10 mM Tris-HCl pH 8, 1 mM EDTA, 250 mM LiCl, 1% NP-40, 1% sodium deoxycholate), and 1 mL TE pH 8.

DNA was eluted twice with 100 µL elution buffer B (50 mM Tris-HCl pH 7.5, 1% SDS, 10 mM EDTA) for 10 min at 60 °C, and 200 µL of TE, pH 8, was added. IP and input samples were reverse cross-linked for 5 h at 65 °C in the presence of 300 µg/mL proteinase K, extracted twice with phenol–chloroform–isoamyl alcohol and once with chloroform, then precipitated at −20 °C with 3.5 µg glycogen.

Final pellets were resuspended in 200 µL H_2_O for IPs and 300 µL for inputs. Samples were validated by qPCR using *oprD* and *czcR* promoter enrichment under CzcR-inducing conditions. Biological triplicates were pooled, and 50 µL of each pooled IP sample was submitted to Fasteris for sequencing (https://www.fasteris.com/en-us/). Replicates were pooled prior to analysis to enhance signal detection, at the expense of assessing inter-replicate variability. Chip-seq libraries were prepared using the TruSeq SBS Kit v3 and sequenced on an Illumina HiSeq 2500 in single-end mode (1 × 50 cycles).

### 2.3. ChIPseq Data Analysis

Raw reads were quality filtered with fastp v1.0.1 [31] using -q 25 -u 40 -e 20 -l 50 options, and reads’ quality was assessed with FastQC v0.12.1 (https://github.com/s-andrews/FastQC). After filtering, 5′375′001, 6′082′065 and 5′253′990 reads were obtained for ZS, ZE and control conditions, respectively. Reads’ depth was normalized to 5× for all conditions using rasusa v2.1.1 with a genome size of 6.2 mb [32]. Reads were mapped to the *Pseudomonas aeruginosa* PAO1 reference genome (ASM676v1) using Bowtie2 v2.5.4 [33]. Only uniquely mapping reads were retained for further analysis. Peak calling was performed with MACS3 v3.0.3 [34] using the control condition sample as reference (-q 0.01 --extsize 200 --nomodel -g 6264404), and peaks’ quality was assessed with PhantomPeakQualTools V1.2.2 [35]. Motif analysis was performed with MEME v5.5.8 [36] using the 101 bp regions centered around best quality peaks (−log10(Qvalue) > 20) and a minimum motif width of 3. The motif found was submitted to FIMO v5.5.8 analysis to scan the entire PAO1 genome to find positions matching this motif. For each peak, the position of the closest matching binding motif was reported when present within the 200 bp around the peak summit and with a *p*-value < 0.01 (Table S1). Finally, only peaks located around start codon sites (−800 bp to +100 bp) were kept since most regulatory sites are located closely to their associated gene start codon. Gene functional annotation was performed using Cluster of Orthologous Groups (COG) categories assigned via the eggNOG database [37]. In addition to our data, we added RNA-seq data from Li et al. [38] in order to refine the binding target analysis, and we used their definitions of regulatory groups: Group 1 includes genes induced by Zn^2+^ exclusively via the CzcR-dependent pathway; Group 2 includes genes repressed by Zn^2+^ exclusively via the CzcR-independent pathway; Groups 3 and 4 include genes positively and negatively regulated by Zn, respectively, possibly through CzcR-independent pathways; and Groups 6–8 include genes regulated by Zn^2+^ through a combination of CzcR-dependent and CzcR-independent mechanisms. Most of these steps were implemented in the reproducible pipeline ChIP-seq_bact, available at https://github.com/fmauffrey/ChIP-seq_bact.

## 3. Results

**Figure 1:**
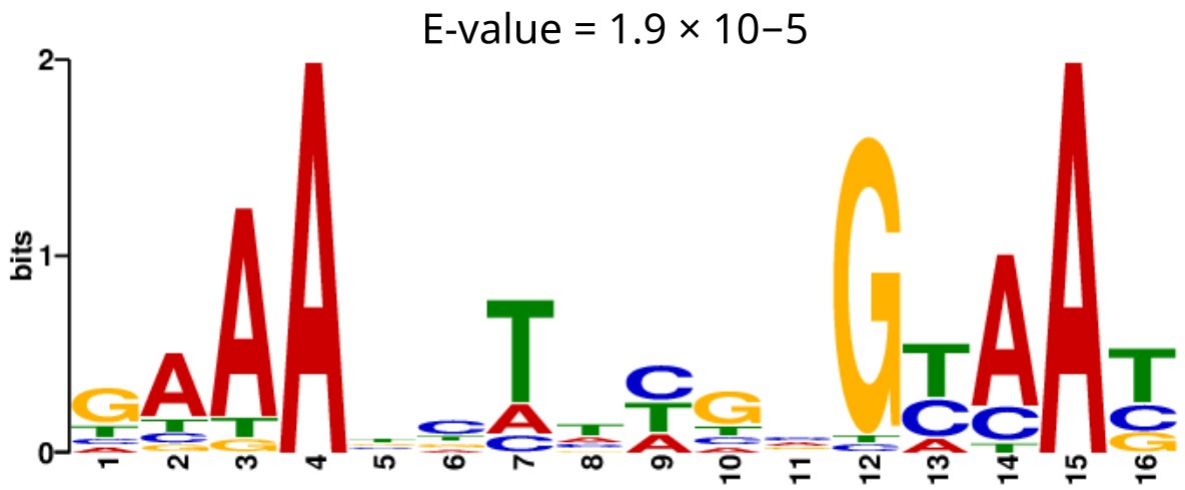
De novo motif identified by MEME using 101 bp sequences centered on the summits of the 36 highest-confidence CzcR binding peaks under ZE conditions. The motif (E-value = 1.9 × 10^−5^) was detected in 34 of 36 sequences used for motif discovery, with letter height indicating nucleotide frequency at each position.

Targeted ChIP experiments using an HA-tagged version of CzcR have previously been performed [18]. To comprehensively characterize the CzcR regulon at the genome-wide level, we conducted ChIP-seq experiments using a polyclonal anti-CzcR antibody in *P. aeruginosa* PAO1 grown under ZS (standard LB medium) and ZE (LB supplemented with 0.5 mM ZnCl_2_) conditions, in order to capture both basal and activated CzcR activity. In addition, a *czcRS* deletion mutant served as a control, enabling high-precision peak calling. Using MACS3, we identified 85 significant peaks for the ZE condition while only 24 peaks were detected in the ZS condition, with fold enrichment varying from 1.6 to 16. For both conditions, these peaks spread all over the *P. aeruginosa* PAO1 genome. To define the CzcR binding motif, we selected high-quality peaks (−log_10_(Q-value) > 20) and submitted 101 bp sequences centered on these peaks to MEME (Multiple EM for Motif Elicitation). Analyses were performed separately for ZS and ZE conditions to detect potential differences in binding motifs. This yielded 36 sequences for ZE and 4 for ZS. For the ZE condition, we found a 16 bp motif (GAAABCTWHGVGYAAY, Figure 1) present in 34 of the 36 submitted sequences with an E-value of 1.9 × 10^−5^. This motif was absent in only two peaks corresponding to PA4142, a putative secretion protein, and *algD* involved in alginate biosynthesis. No significant motif was identified in the ZS condition using MEME analysis, likely due to the limited number of sequences analyzed rather than an absence of a motif, limiting our conclusions on this aspect of the study. The FIMO analysis showed that the motif was significantly present at many positions in the PAO1 genome, indicating widespread potential CzcR binding (Table S1).

**Figure 2:**
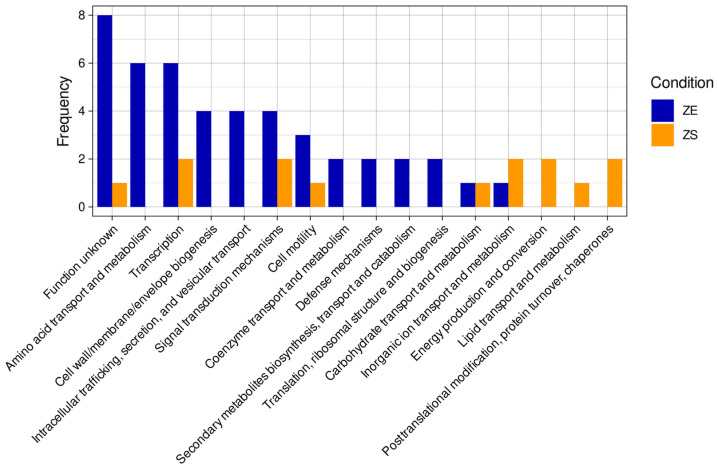
Functional categorization (COG) of CzcR-regulated genes in ZE and ZS conditions.

Most regulator binding sites are located near the transcription start site (TSS) of their target genes; thus, to identify genes potentially regulated by CzcR, we focused on peaks located within −800 to +100 bp of the start codon of nearby genes. This yielded 61 peaks under the ZE condition and 22 peaks under the ZS condition, corresponding to 112 and 37 genes/operons, respectively (Tables S2 and S3). After removing hypothetical proteins, ncRNA and peaks with no close binding motifs found, 32 and 10 peaks were kept for ZE and ZS conditions, respectively (Table 1 and Table 2). Among these, seven genes previously reported to be under CzcR control were identified (*czcRS*, *czcCBA*, *oprD, phzA1*, *phzM*, *phzB2* and *ptrA*) [15,18,22], thus validating our ChIP-seq approach. Under the ZS condition, no known CzcR-regulated genes were detected. Overall, peak fold enrichment (ratio of protein binding at a specific DNA region in the experimental condition compared to the control) was higher in the ZE condition (mean = 4.6) than in the ZS condition (mean = 2.1). Only two genes were common to both conditions: *roxS* (sensor histidine kinase) and PA0178 (two-component sensor). Fold enrichment was comparable between conditions for both genes. Under ZS conditions, most genes potentially regulated by CzcR belonged to various categories while the most represented categories were “Amino acid transport and metabolism” and “Transcription” under the ZE condition. (Figure 2, Table S4). Notably, several COG categories were differentially represented between conditions, with some categories present under ZE conditions but absent under ZS conditions, and vice versa. Additionally, we incorporated transcriptomic data from a previous study on PAO1 grown under similar conditions [38] to extend our analysis (Table 1 and Table 2). In order to extract genes with a high probability to be regulated by CzcR, we considered peaks with a binding motif very close to the peak summit (<15 bp between peak summit and the center of the binding motif) and having a potential direct regulation by CzcR (from RNA-seq data). Following this approach, we identified seven genes under the ZE condition that have not been previously described: PA4500, PA4498, *pvdS*, *pvdG*, *opdT*, *himA* and *piv*. Using the same approach, we identified four genes under ZS condition: *bfrB*, *cspD*, PA5436 and PA5437. All of these newly identified targets were also supported by RNA-seq data [38], providing independent evidence of transcriptional regulation; however, these genes should still be considered as putative targets pending direct functional validation. The two genes potentially regulated by CzcR in both ZE and ZS conditions (*roxS* and PA0178) were not included in these lists since their respective closest binding motifs were relatively far (>50 bp) from the peak summit.

## 4. Discussion

In this study, we aimed to improve the mapping of the CzcR regulon in *P. aeruginosa* under conditions of ZS concentration or ZE concentration. These results extend previous efforts to characterize CzcR-dependent regulation and provide a broader genome-wide perspective on its activity under distinct Zn conditions. Using a ∆*czcRS* double mutant as a control in our ChIP-seq experiment, we ensured that peak detection was as accurate as possible. Using peaks presenting the highest quality, a 16 bp motif was identified as the CzcR binding motif (GAAABCTWHGVGYAAY) and was similar to the motif (GAAAC-N6-GTAAT) already described in another study [27]. These findings confirm that CzcR binds to a specific motif within target promoters under activation conditions. Under the ZE condition, 32 significant peaks were detected, representing 39 potentially regulated genes. In their study, Fan et al. [27] identified 16 significant peaks as CzcR binding sites. Differences in filtering criteria and use of input DNA as a control likely account for this discrepancy. Ten peaks from the Fan dataset were also identified here, while six others were excluded because no binding motif was found close to the peak summit (three peaks) or because a corresponding peak was present in the control condition (three peaks). We identified 10 binding sites of CzcR under ZS conditions, potentially regulating 13 genes, reinforcing the idea of basal activity. For most of these peaks, a weaker binding signal was also present for the control and ZE conditions, suggesting potential nonspecific binding at these sites. These peaks exhibited low fold enrichment values (1.7 to 2.6), indicating that CzcR binds with lower affinity compared to the fully activated form. Moreover, some significant peaks exhibited low raw signal intensity, suggesting they may arise from background noise. These observations introduce limitations, making it challenging to draw definitive conclusions about these peaks. Nevertheless, low-affinity interactions may still be biologically meaningful, as they could be stabilized by genome accessibility, nucleoid-associated proteins, heterodimerization with other transcription factors, or other co-regulatory mechanisms. Surprisingly, in neither ZE nor ZS conditions, *lasI* was not detected among these targets, suggesting that its regulation may be context-dependent or state-dependent, consistent with its prior detection using a tagged form of CzcR [18]. Multiple lines of evidence may suggest that this basal activity derives from an intrinsic ability of CzcR to bind DNA even in the absence of phosphorylation. The motif analysis indicated that most of these basal targets displayed a close CzcR binding motif with a high *p*-value (<0.01), suggesting that unphosphorylated CzcR could bind to a similar binding motif. Functional annotation of the genes identified under ZS and ZE conditions revealed differences in the biological processes associated with each condition. In particular, ZE-associated targets were mainly related to zinc homeostasis, transport, and virulence-associated functions, whereas ZS-associated targets were more consistent with basal adaptive and survival-related processes. These observations suggest that CzcR regulatory output may differ depending on zinc availability. Moreover, the basal regulon suggests that CzcR contributes to processes distinct from metal detoxification. Altogether, these pathways highlight a role for basal CzcR activity in maintaining physiological robustness, regulation functions and stress response, albeit at lower affinity compared to the activated form. Together, these findings highlight the complexity of CzcR-mediated regulation and suggest that its activity is not restricted to zinc detoxification but may also contribute to broader adaptive and virulence-related processes.

By filtering our ChIP-seq results to keep only peaks with a very close CzcR binding motif (<15 pb) and by integrating transcriptomic data from another study, we identified seven new genes (*dppA3*, *mdpA*, *pvdS*, *pvdG*, *opdT*, *himA* and *piv*) with a high probability of being directly regulated by CzcR under the ZE condition [22]. While *dppA3* and *opdT* are associated with molecular transport, the remaining five genes (*mdpA*, *pvdS*, *pvdG*, *himA* and *piv*) are integral to the *P. aeruginosa* virulence repertoire: *mdpA* mediates cellular cytotoxicity, *pvdS* and *pvdG* are essential for pyoverdine biosynthesis, *himA* regulates alginate production, and *piv* functions as a virulence factor [39,40,41,42,43,44,45]. Interestingly, *opdT* has been proven to be regulated by ZnO microparticles, and, given these new insights, this mechanism likely occurs under the control of the CzcRS pathway [43]. These findings further underscore the intricate link between zinc homeostasis and virulence in *P. aeruginosa* [5]. This observation reinforces the emerging view that metal homeostasis regulators can directly influence virulence-associated pathways in *P. aeruginosa*.

Under the ZS condition, four genes were identified as highly likely regulated by CzcR. As observed under the ZE condition, two of these genes (*pycA* and *pycR*) are implicated in bacterial virulence, specifically through their involvement in the production of the virulence factor pyruvate carboxylase [46]. The remaining two genes, *bfrB* and *cspD*, are associated with bacterial survival strategies: *bfrB* mediates iron storage, while *cspD* functions as a cold-shock response protein [47,48].

In this study, we further explored the CzcR regulon by providing new insights into the genes potentially controlled under both ZE (activated form) and ZS (basal activity) conditions. Overall, our findings support a model in which CzcR may exert both zinc-dependent and basal regulatory functions. While these observations are supported by combined ChIP-seq and transcriptomic evidence, the newly identified targets remain putative and will require further experimental validation to confirm direct regulatory interactions. Understanding these mechanisms will provide a more comprehensive view of how *P. aeruginosa* integrates metal sensing with antibiotic resistance and virulence.

## Figures and Tables

**Table 1:** Significant peaks identified in the ChIP-seq analysis under the ZE condition. Genes likely under direct CzcR regulation and not previously described are indicated in bold. Regulatory groups are defined as in the study by Li et al. [38]: Group 1 includes genes induced by Zn^2+^ exclusively via the CzcR-dependent pathway; Group 2 includes genes repressed by Zn^2+^ exclusively via the CzcR-independent pathway; and Groups 6–8 include genes regulated by Zn^2+^ through a combination of CzcR-dependent and CzcR-independent mechanisms. * *p*-value < 0.01, ** *p*-value < 0.001 and *** *p*-value < 0.0001.

Peak	Distance Between Peak Summit and Center of the 16 bp Binding Motif (bp)	*p*-Value Binding Motif FIMO	Locus	Gene	Product	Referenced CzcR Regulation	RNA-Seq Data ^4^
6	1	***	PA0500	*bioB*	Biotin synthase		
			PA0499	PA0499	Pili assembly chaperone		
18	14	***	PA0958	*oprD*	Porin D	Repression ^1^	Group 2
56	21	***	PA2808	*ptrA*	Repressor PtrA	Induction ^2^	Group 1
			PA2809	*copR*	Two-component response regulator CopR		
62	43	***	PA3280	*oprO*	Pyrophosphate-specific outer membrane porin OprO		Group 8
72	3	***	PA4138	*tyrS*	Tyrosine–tRNA ligase		
75	6	***	PA4210	*phzA1*	Phenazine biosynthesis protein	Repression ^1^	
			PA4209	*phzM*	Phenazine-specific methyltransferase	Repression ^1^	Group 7
3	9	**	PA0291	*oprE*	Anaerobically induced outer membrane porin OprE		Group 2
21	4	**	PA1004	*nadA*	Quinolinate synthetase		
		**	PA1003	*mvfR*	Transcriptional regulator MvfR		
22	8	**	PA1113	PA1113	ABC transporter ATP-binding protein/permease		
24	38	**	PA1151	*imm2*	Pyocin-S2 immunity protein		Group 8
30	30	**	PA1382	PA1382	Type II secretion system protein		
47	7	**	PA2522	*czcC*	Outer membrane protein CzcC	Induction ^3^	Group 1
			PA2523	*czcR*	Two-component response regulator	Induction ^1^	Group 1
50	40	**	PA2592	PA2592	Spermidine/putrescine-binding protein		
51	23	**	PA2696	PA2696	Transcriptional regulator		
58	27	**	PA3141	*wbpM*	Nucleotide sugar epimerase/dehydratase WbpM		
68	3	**	PA3540	*algD*	GDP-mannose 6-dehydrogenase AlgD		
78	40	**	PA4494	*roxS*	Sensor histidine kinase RoxS		
79	12	**	PA4500	*dppA3*	ABC transporter		Group 8
			PA4498	*mdpA*	Metallopeptidase		Group 8
1	39	*	PA0178	PA0178	Two-component response regulator		Group 8
35	33	*	PA1900	*phzB2*	Phenazine biosynthesis protein PhzB	Repression ^1^	Group 2
36	1	*	PA1971	*braZ*	Branched-chain amino acid transport system 3 carrier protein		
38	3	*	PA2042	PA2042	Serine/threonine transporter SstT		Group 7
44	13	*	PA2426	*pvdS*	Extracytoplasmic-function sigma-70 factor		Group 7
			PA2425	*pvdG*	Pyoverdine biosynthesis protein PvdG		Group 7
46	7	*	PA2505	*opdT*	Tyrosine porin OpdT		Group 6
49	53	*	PA2570	*lecA*	PA-I galactophilic lectin		Group 8
52	36	*	PA2698	PA2698	Hydrolase		
53	25	*	PA2735	PA2735	Restriction-modification system protein		
54	6	*	PA2738	*himA*	Integration host factor subunit alpha		Group 8
60	21	*	PA3153	*wzx*	O-antigen translocase		
73	48	*	PA4142	PA4142	Secretion protein		Group 7
74	1	*	PA4175	*piv*	Endopeptidase IV		Group 1
84	16	*	PA5170	*arcD*	Arginine/ornithine antiporter		

^1^ Dieppois et al. [18]; ^2^ Li et al. [22]; ^3^ Perron et al. [15]; ^4^ Li et al. [38].

**Table 2:** Significant peaks identified in the ChIP-seq analysis under the ZS condition. Hypothetical proteins and ncRNA were also removed. Genes likely under direct CzcR regulation and not previously described are indicated in bold. Regulatory groups are defined as in the study by Li et al. [38]: Groups 5–8 include genes regulated by Zn^2+^ through a combination of CzcR-dependent and CzcR-independent mechanisms. * *p*-value < 0.01, ** *p*-value < 0.001 and *** *p*-value < 0.0001.

Peak	Distance Between Peak Summit and Center of the 16 bp Binding Motif	FIMO *p*-Value for Binding Motif	Locus	Gene	Product	RNA-Seq Data ^1^
18	8	***	PA3531	*bfrB*	Bacterioferritin	Group 8
			PA3529	PA3529	Peroxidase	
20	54	**	PA4494	*roxS*	Sensor histidine kinase RoxS	
1	51	*	PA0178	PA0178	Two-component sensor	Group 8
2	54	*	PA0195	*pntAA*	NAD(P) transhydrogenase subunit alpha	
7	54	*	PA1317	*cyoA*	Cytochrome o ubiquinol oxidase subunit II	
11	13	*	PA2621	PA2621	ATP-dependent Clp protease adapter protein Clp	
			PA2622	*cspD*	Cold-shock protein CspD	Group 8
14	31	*	PA3190	PA3190	Sugar ABC transporter substrate-binding protein	Group 7
15	41	*	PA3279	*oprP*	Phosphate-specific outer membrane porin OprP	
22	39	*	PA4891	*ureE*	Urease accessory protein UreE	
24	7	*	PA5436	*pycA*	Acetyl-CoA carboxylase subunit alpha	Group 5
			PA5437	*pycR*	Transcriptional regulator	Group 7

^1^ Li et al. [38].

## Data Availability

The CzcR ChIPseq data files were deposited in the European Nucleotide Archive and are accessible through the project accession number PRJEB104501.

## Supplementary Materials

The following supporting information can be downloaded at https://www.mdpi.com/article/10.3390/microorganisms14050943/s1, Table S1: Results of the FIMO analysis using the binding motif determined by the MEME analysis; Table S2: Peaks from the MACS3 analysis under the ZE condition; Table S3: Peaks from the MACS3 analysis under the ZS condition; Table S4: Functional classification of genes by COG.

## Abbreviations

The following abbreviations are used in this manuscript:

ZS	Zinc Sufficiency
ZE	Zinc Excess
TCS	Two-Component System
COG	Cluster of Orthologous Groups
